# A 2.5° × 2.5° gridded drought/flood grades dataset for eastern China during the last millennium

**DOI:** 10.1038/s41597-023-02110-5

**Published:** 2023-04-11

**Authors:** Zhixin Hao, Jingyun Zheng, Quansheng Ge, Mengxin Bai

**Affiliations:** 1 Key Laboratory of Land Surface Pattern and Simulation, Institute of Geographic Sciences and Natural Resources Research, Chinese Academy of Sciences, Beijing, 100101 China; 2 University of Chinese Academy of Sciences, Beijing, 100049 China; 3 Beijing Municipal Climate Center, Beijing Meteorological Bureau, Beijing, 100089 China

**Keywords:** Palaeoclimate, Climate change

## Abstract

Hydroclimate reconstruction for the last millennium is essential to understand the differences in hydroclimate extremes and their causes under cold/warm conditions. In this study, the first gridded drought/flood grades (D/F grades) dataset in eastern China (EC) during the last millennium was generated. This D/F grades dataset mainly consisted of two components. The first section was created by interpolating drought/flood grades from 1500 to 2000 using the angular distance weight method. Sampling error estimates were employed to assess the effects of the interpolated dataset. The second section for the D/F grades dataset during 960–1500 was generated by constructing best subset regression models using selected tree-ring chronologies in the United States through atmospheric teleconnection. The validation parameters of the calibration equations were also derived, including the adjusted *R*^*2*^, predicted *R*^*2*^, RE, and CE. This dataset provides critical support for investigating the characteristics and causes of hydroclimate extremes in EC at various spatiotemporal scales, as well as the relationship with climate modes, such as El Niño-Southern Oscillation, Pacific Decadal Oscillation, and East Asia Summer Monsoon.

## Background & Summary

Increasing hydroclimate anomalies have aroused widespread concern for their extensive effects on ecosystems and societies^1–3^. The World Meteorological Organization (WMO) reported that hydroclimate-related disasters reaching ~50% have dominated natural disasters since the 1970s^4^. For instance, heavy rainfall with a 1000-year return period in Germany on July 2021, has caused over 135 deaths^5^. The strongest mountain fires sparked by Australia’s compound drought in 2019–2020 killed at least 33 people and 1 billion animals^6^. Moreover, similar extreme hydroclimates have been detected across Eurasia and North America, and local agricultural and ecological droughts have further increased^7,8^.

The hydroclimate variations in eastern China (EC) are closely related to the intensity of the Asian summer monsoon^9–11^. Hydroclimate extremes induced by monsoon systems have caused serious economic losses in EC. For instance, extreme droughts in 2010 and 2019 devastated Southwest China, resulting in a total of USD 70 billion losses^12,13^. A record-breaking Meiyu rainfall event in the Yangtze-Huaihe River valley (YHRV) in 2020 caused 141 deaths and USD 11.76 billion economic losses^14,15^. Most importantly, EC will face increasing risks of population exposure to frequent hydroclimate extremes in the future^16–18^. By the end of the 21^st^ century, the intensity of hydroclimate extreme will increase by 0.2 and 0.5 times under low- and high-emission scenarios, respectively^19,20^. Thus, improving the ability of hydroclimate prediction in climate models is important for reducing disaster risks and understanding ecosystem degradation and recovery. However, a reliable and long-term dataset with high temporal and spatial resolutions is necessary to support accurate predictions of climate models.

Regional hydroclimate series (e.g., drought/flood and precipitation) over the past several hundred years to millennia have been reconstructed using multiple proxy data (i.e., historical document, tree ring and ice core) for EC^21–24^. Additionally, some gridded hydroclimate reconstructions have been established. For example, Cook *et al*. (2010)^25^ reconstructed the gridded Asian summer (June-August) Palmer drought severity index (PDSI) for the past 700 years based on tree-ring chronologies throughout the whole region. Moreover, combined with historical document derived from EC, two sets of warm-season (May-September) precipitation gridded data for the Asian continent were produced for the past approximately 500 years^26,27^, while the latest gridded dataset by Shi *et al*. (2018)^28^ was generated for Asian summer precipitation (June-August) during the past 500 years. These proxy-based high-resolution reconstructions provided basic datasets for investigating extreme hydroclimatic events on long-term scales^29,30^. Furthermore, several extreme droughts in EC have been identified, i.e., 1637–1643 in the late Ming Dynasty and 1876–1878 in the Qing Dynasty, which have never been recognized in the instrumental measurement period^31–33^. However, our knowledge about the dryness/wetness variations in EC for the last millennium covering the Medieval Climate Anomaly, Little Ice Age and Current Warm Period, is still very poor due to the very limited proxy data available in this region^34,35^, and it is insufficient to understanding the mechanism of hydroclimate extremes between the cold and warm climatic conditions. Therefore, a well-calibrated, high temporal-resolution reconstruction dataset for the last millennium is essential for studying hydroclimate extremes and their linkages with large-scale circulation. This new millennial-scale dataset could be used to study the interdecadal-centennial-scale characteristics of hydroclimate variations and extreme hydroclimatic events under the different backgrounds of cold/warm climates and their relationships with climatic modes. Additionally, it could be also used to assess the ability of the model to simulate climate for the last millennium and diagnose mechanisms of climate change.

This work generated the first millennial EC drought/flood grades (D/F grades) dataset with 2.5° × 2.5° grid point spatial resolution and yearly temporal resolution. Here, we used 76 annually resolved historical document-based drought/flood grades data from 960 to 2000 in EC; 5292 annually resolved tree ring chronologies (TRCs) and one speleothem chronology with a 3–5-year resolution in Dayu Cave (106.3°E, 33.13°N). The reconstruction processes of this dataset consisted of two parts. The first part involved developing the gridded dataset from 1500 to 2000 by interpolating drought/flood grades using the angular distance weight method. The second part involved gridded dataset reconstruction from 960 to 1500 by conducting calibration models by TRCs using best subset method. Compared with the other three existing high-confidence gridded datasets, our dataset extended forward by at least 500 years. Moreover, the new dataset’s explained variance (R^2^) was significantly better than those in Feng (2013) and Shi (2018), improving by approximately 0.2, similar to Shi (2017). Here, we provide a thorough validation of all the reconstructed variables and include an uncertainty estimation for the reconstructed hydroclimate.

## Methods

### Grid division

This study region is in eastern China and spatially covers 103.75°E–125°E and 18.75°N–41.25°N. EC is a central region controlled by Asian Monsoon, and its summer precipitation variability is significantly dominated by the intensity of East Asian Summer Monsoon, where water vapor is transported from the western Pacific Ocean and the Bay of Bengal. The spatial patterns of precipitation variation in EC are usually illustrated latitudinally with three regions of North China, Yangtze-Huaihe River valley and South China in the previous study^36^. The 76 historical drought/flood grades (DFG) sites are distributed evenly in EC, and to improve the reliability of hydroclimate reconstruction, at least one proxy is assured for each grid and each year. Thus, the spatial resolution of the gridded dataset is set as 2.5- by 2.5-degrees.

### Reconstruction method

**Step 1: Interpolation and error estimation for 1500**–**2000 CE**

Since there were no missing DFG data from 1500 to 2000, the angular distance weight (ADW)^37^ interpolation was utilized to develop a gridded D/F grades dataset in this study. Compared with several interpolation methods, including thin-plate spline, triangulation, and Thiessen polygon interpolation, we found that ADW fully utilizes hydroclimate information for all stations and employs a distance weighting function to give the stations nearest to grid points more weight. Moreover, ADW interpolation constrains the relative contribution of different stations based on the linkage of the angular weight among stations. This method has been extensively applied in climate interpolation, such as in Climate Research Unit (CRU) gridded climate datasets. The first component, distance weight (*w*_*k*_), is generally defined by Jones *et al*. (1997)^38^ as Eq. (1).

1 $${w}_{k}={e}^{-xm/{x}_{0}}$$

where *x*_*0*_ is the correlation decay distance (CDD), *x* is the distance from the station to the center of the grid point, and *m* is a constant. Precipitation variations are highly spatially heterogeneous and spatial correlations of precipitation between one grid and others decay rapidly outward from the grid center^36,39^, and the precipitation’s CDD at the midlatitude regions over the NH is approximately 450 km. Here, the CDD is set as 400 km to improve the reliability of spatial interpolation. We calculated the distance between all stations and each grid center first, and then those stations within a 400 km distance were selected as alternative stations for the corresponding grid. The criterion of CDD less than 400 km could ensure at least one station for each grid to be used for hydroclimatic interpolation and even one grid (e.g., 115°E, 35°N) contained 16 stations. A previous study confirmed that a lower value for *m* resulted in too much smoothing while higher value reduced the impact of more distant points on the grid, and multiple cross-validation revealed that a value of 4 for *m* produces the lowest error^36^. The second component, angular weight (*a*_*k*_), was determined by the angular isolation of each *n*_*j*_ grid point selected in Eq. (2).

2 $${a}_{k}=\mathop{\sum }\limits_{l=i}^{{n}_{j}}{w}_{l}(1-cos{\theta }_{j}(k,l))/\mathop{\sum }\limits_{l=1}^{{n}_{j}}{w}_{l}\quad \quad l\ne k$$

where *θ*_*j*_*(k, l)* is the angular separation of stations *k* and *l*, with the vertex of the angle defined at grid point *j*, which is calculated in spherical coordinates; and *w*_*l*_ is the distance weight at station *l* to grid *j*. The distance weight and angular weight are then combined into an angular-distance weight by Eq. (3).

3 $${W}_{k}={w}_{k}\left(1+{a}_{k}\right)$$

The D/F grades dataset for 1500–2000 CE was interpolated by the angular distance weight method. Moreover, errors from interpolation were dependent on the number and distance to the grid center of selected stations for each grid. Therefore, the sampling error estimation method was used to the evaluate error range. Although the true value of the standard deviation ($\widehat{S}$) of the interpolated result could only be the average standard deviation (*S*_*i*_) of countless sampling station series, the numbers of observational stations are limited. $\widehat{S}$ is related not only to *S*_*i*_ of the selected stations, but also to the interstation correlation coefficient ($\bar{r}$). Therefore, $\widehat{S}$ is generally estimated by Eq. (4). Of course, a higher station density would possibly lead to better estimates^38^.

4 $${\widehat{S}}^{2}={\bar{S}}_{i}^{2}\left[\left(1-\bar{r}\right)/\left(1+\left(n-1\right)\bar{r}\right)\right]$$

Here, we assumed that the estimates of ${\bar{S}}_{i}^{2}$ and $\bar{r}$ are unbiased and could yiled *S*^2^ by letting n → ∞ in Eq. (4), and then Eq. (5) is deduced as

5 $${S}^{2}={S}_{i}^{2}\bar{r}$$

Based on the unbiased estimation hypothesis, Eq. (5) gives a relationship between the standard deviation of stations’ hydroclimate series and the average correlation coefficient among stations. Note that *S*^2^ is independent of the number of stations selected from the gird points. Thus, Eq. (5) with ${\bar{S}}_{i}^{2}$ and $\bar{r}$ could be estimated by stations’ hydroclimate information. Finally, the sampling error (SE) estimation was calculated by Eq. (6).

6 $${{\rm{SE}}}^{2}={\bar{S}}_{i}^{2}\bar{r}\left(1-\bar{r}\right)/\left(1+\left(n-1\right)\bar{r}\right)$$

where ${\bar{S}}_{i}^{2}$ is the average of the variance for all station series in the reference period (i.e., 1961–1990 CE). The station number selected by each grid interpolation process is rarely constant. It is preferable to estimate the average variance (i.e., ${\bar{S}}_{i}^{2}$) of the series from the selected stations inside the grid using the variance of the grid series, which allows errors in station density and location changes over time to be ignored. Moreover, $\bar{r}$ is estimated among station series, and the estimation of $\bar{r}$ is valid for a grid that includes the multiple stations. However, when very few stations are available in the grid, the confidence of $\bar{r}$ is relatively low; in particular, for one station within a grid point, the value cannot be estimated. Therefore, the decay length theory of precipitation correlation is used to estimate $\bar{r}$ in Eq. (7).

7 $$\bar{r}=\left({x}_{0}/x\right)\left(1-{e}^{-x/{x}_{0}}\right)$$

where *x* is the distance of the diagonal grid in this study, i.e., 37.08 km, and *x*_*0*_ is the CDD, i.e., 400 km, then, the estimated value of $\bar{r}$ is 0.955.

**Step 2: Correlation analysis of TRCs and hydroclimate**

Because of the many missing values for DFG data in EC during 960–1500, the ADW method cannot be used for developing the D/F grades dataset. Thus, we attempted to find possible linkages between hydroclimate variations in EC and tree-ring width/density chronologies in the NH from the atmospheric teleconnection perspective and further generated regression models that can be used for hydroclimate reconstruction. To find TRCs that can well reflect local precipitation variations, the correlations between TRCs over the NH and local summer precipitation during 1921–1980 were first calculated. And, above-mentioned chronologies in NH with significant positive correlations (*p* < 0.05) were selected. Generally, we regarded these TRCs could reflect local precipitation/hydroclimate variations. Then, TRCs which were selected over the NH, were also assigned into different regions (mostly in United States). For constructing calibration equations between the TRCs in other regions over NH and the hydroclimate variations in EC, it is necessary to find TRCs that could reflect hydroclimate variations of EC. So, the next step was to identify hydroclimate teleconnection between other regions over NH and EC in order to further screen the TRCs.

**Step 3: Model validation and selection**

We examined the spatial teleconnection patterns of hydroclimate variation between EC and United States where TRCs were chosen in step 2 in order to show that these selected TRCs in above-mentioned could be used to reconstruct the hydroclimate variation in NC. The singular value decomposition (SVD) method was first performed to reveal the first mode spatial patterns of precipitation in EC with the regions where the chosen TRCs were located using precipitation data from GPCC in 1921–2000. Then, the SVD analysis was carried out to reveal the first mode spatial patterns of precipitation between EC and the regions where the chosen TRCs were located using precipitation data from a total of 13 ensemble cases of CESM-LME in 1921–2000. Through comparing the spatial patterns of the observations with the patterns, we selected the patterns that can reproduce the spatially remote correlation patterns revealed by the observations. We chose these cases in CESM-LME that could reproduce the spatial teleconnection patterns by comparing the spatial patterns of the observations and simulations. These simulation results had a superior ability to capture the observed facts.

**Step 4: Diagnostic analysis of the teleconnection pattern**

By comparing spatial patterns of teleconnection between observation and simulation in step 3, we selected the optimal model cases that could better reproduce their spatial patterns. Due to the systematic errors in the climate model, the results of multi-model cases ensemble mean (MME) in this study were used to diagnose the spatial patterns of teleconnection and their potential mechanisms in EC and the United States from 960 to 1500. Using precipitation data from MME results of CESM-LME between 960–1500, the SVD analysis was first conducted to reveal the spatial patterns of first mode between EC and the United States. Then, the spatial regression coefficients between the time series of the first mode of EC and the United States and SST anomalies were respectively calculated to determine the sensitivity of SST variations to hydroclimate in EC and the United States. Next, spatial regression coefficients of the time series of the first mode with the geopotential height anomaly of the 500 hPa pressure level, zonal wind anomaly of the 200 hPa pressure level and wind anomaly of the 850 hPa were calculated to identify the hydroclimate teleconnection between EC and the United States from the perspective of large-scale atmospheric circulation. Finally, spatial regression coefficients of the time series of the first mode with the vertical wind anomaly of the 500 hPa pressure level were calculated to reveal the potential mechanism of the teleconnection of hydroclimate variations in the above-mentioned two regions from the perspective of the vertical circulation.

**Step 5: Regression model and validation during 960**–**1500 CE**

Due to different lengths of tree-ring chronologies and years of missing DFG data, proxy data from the same period were utilized for reconstructing the calibration equation in the corresponding period. In detail, for each special period, the best regression model with maximum ${R}_{a}^{2}$ values produced by the best subset selection (BSS) was used for building the calibration equation, and the calibration period was 1921–1980. The number of independent variables in the best regression model was inconsistent in different periods. A higher ${R}_{a}^{2}$ was able to effectively eliminate the effect of changes in the degrees of freedom of the independent variables in different periods. As the reconstructions were calibrated from different equations with different variances, the magnitudes of the reconstructed hydroclimate had to be adjusted using the variance matching method^40^. The method of variance matching calibrated and articulated several reconstructed hydroclimate series from different equations with the ${R}_{a}^{2}$ in the calibration period (1921~1980) as a reference. As a result, an annual homogeneous D/F grades reconstruction dataset was developed.

In this study, three statistical parameters, the reduction of error (RE), coefficient of efficiency (CE), and predicted R-squared (${R}_{pr}^{2}$), were employed to verify the stability of the calibration equation. RE and CE were calculated for the cross-calibration and validation in the periods of 1921–1950 and 1951–1980, respectively, with Eqs. (8, 9).

8 $${\rm{RE}}=1-{\sum }_{i=1}^{n}{({X}_{i}-\widehat{{X}_{ic}})}^{2}/{\sum }_{i=1}^{n}{({X}_{i}-\bar{{X}}{}_{c})}^{2}$$

9 $${\rm{CE}}=1-{\sum }_{i=1}^{n}{({X}_{i}-\widehat{{X}_{iv}})}^{2}/{\sum }_{i=1}^{n}{({X}_{i}-\bar{{X}}{}_{v})}^{2}$$

where *n* is the length of the calibration or validation period; *X*_*i*_ is the observation at year *i*; $\widehat{{X}_{ic}}$ and $\widehat{{X}_{iv}}$ are the predicted values of the regression model in year *i* in the calibration period and validation period, respectively; and $\bar{{X}}{}_{c}$ and $\bar{{X}}{}_{v}$ are the average hydroclimates during the calibration period and validation period, respectively. Generally, the regression equation with the BSS method is stable and reliable, when CE and RE are greater than zero^41^. In addition, ${R}_{pr}^{2}$ was calculated for the calibration equation in the period of 1921–1980 with Eq. (10).

10 $${R}_{pr}^{2}=1-{\sum }_{i=1}^{n}{({X}_{i}-\widehat{{X}_{i}})}^{2}/{\sum }_{i=1}^{n}{({X}_{i}-\bar{{\rm{X}}})}^{2}$$

where *n* is the length of the calibration equation, which is 60 in this study; *X*_*i*_ is the observational value in year *i*; $\bar{X}$ is the average value in the calibration equation; and $\widehat{{X}_{i}}$ is the predicted value of the regression model in year *i* in the calibration equation.

One grid (115°E, 35°N) is used as an example to show the reconstruction process. Its alternative proxies include 16 DFGs and 4 TRC data. The best regression model was first generated by the BSS method. A total of 8 DFGs and 4 TRCs was selected as candidate independent variables in 960–961 CE. Its statistical parameters of the calibration equations of ${R}_{a}^{2}$ reached 0.83. Then, during the period of 962 CE, 1010 CE, and 1016 CE, etc., available proxies are consistent, and the best calibration equation was constructed by the BSS method. A total of 8 DFGs and 3 TRCs was selected as candidate independent variables. Its statistical parameters of the calibration equations of ${R}_{a}^{2}$ was 0.85. According to this approach, the best calibration equations were constructed in the period with different numbers of available proxies, which are shown in Table S1. Importantly, the variance explained by the calibration equations in different periods differed. Using the highest ${R}_{a}^{2}$ as a reference, the variance matching method was used to calibrate the hydroclimate reconstruction in different periods to form a homogeneous hydroclimate dataset with annual resolution.

## Data Sources

The data used in this study consist of proxy data, instrumental data and simulation data. Three sets of proxy datasets include drought/flood grades data from Chinese historical documents, tree-ring width chronologies and tree-ring density chronologies over the NH, and speleothem chronology in EC. The monthly gridded precipitation dataset is available from the Global Precipitation Climatology Center. And, the simulation results from the Community Earth System Model Last Millennium Ensemble experiment (CESM-LME) are used.

### Proxy data

**Fig. 1:**
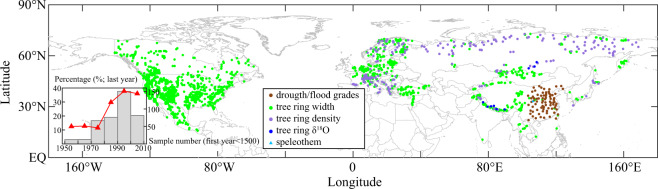
Spatial distribution of DFG sites and tree-ring sample sites over the NH. The percentage of tree-ring chronologies by the ending years to the total and number of tree-ring-based proxies longer than 500 years are shown in the left-bottom corner. (Brown circles represent historical document based-drought/flood grades; green circles represent tree ring width chronologies; purple circles represent tree ring density chronologies; blue circles represent tree ring δ^18^O chronologies; green triangles represent speleothem chronologies).

**Fig. 2:**
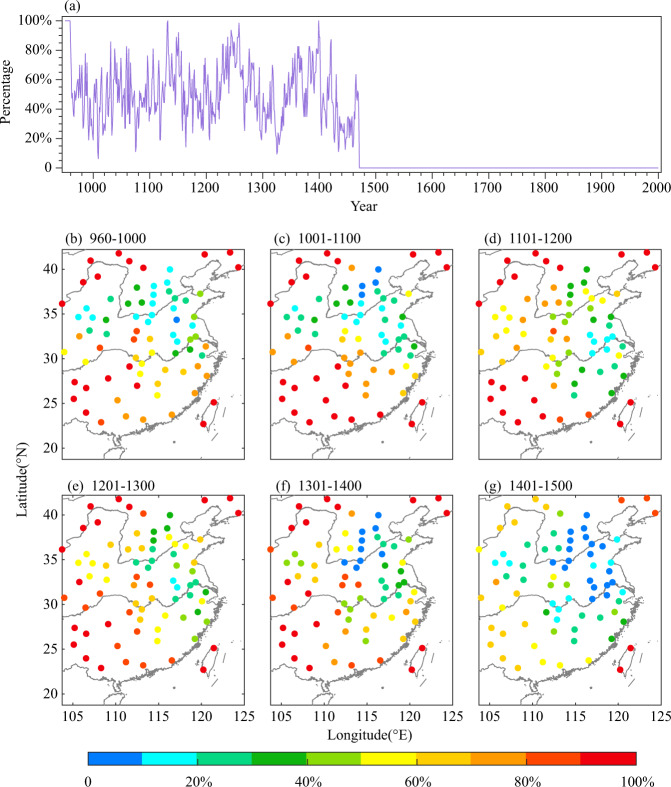
Temporal variations in the rate of missing DFGs for all EC sites (**a**) and the spatial distribution of the rate of missing DFGs per 100 years (**b**–**g**).

The drought/flood grades showed hydroclimate variations for 76 stations in EC during the last millennium with an annual resolution^21^ (Figs. 1, 2). They were derived from descriptions of the location, intensity and duration for drought and flood disasters and usually recorded in Chinese historical documents, along with direct impacts on agriculture and society. Based on historical documents, the intensity and duration of drought and flood disasters in each region were quantified into five grades: 1 (large flood), 2 (flood), 3 (normal), 4 (drought), and 5 (severe drought), with probabilities of occurrence of 10%, 25%, 30%, 25%, and 10%, respectively^42^. The documents used for DFGs can be divided into three categories: (1) Jing, Shi, Zi and Ji (the Classics, Histories, Schools, and Collections) from the mid-*Xi-han* Dynasty to the mid-*Ming* Dynasty (137 BCE-1470 CE);^43^ (2) official histories and local gazettes from the mid-*Ming* Dynasty to the Republic of China (1470 CE-1949 CE); (3) official archives and newspapers from the Qianlong emperor of Qing Dynasty to the Republic of China (1736 CE-1949 CE). Due to the differences in original recording sources and characteristics in different periods, the DFG dataset used different quantitative methods, i.e., the grading of literature descriptions before 1470, with stated the frequencies of counties with droughts and floods at each site from 1470 to 1949, and after 1949, using the criteria of 0.33 times and 1.17 the times standard deviation (basically equal to the probability distribution of DFGs in the historical period) to grade summer precipitation. Finally, the reconstructions from the three periods were calibrated and merged into a homogenous DFG dataset using Fisher’s linear discriminants and Bayesian estimates throughout the entire time series for each station^21^. More historical documents were missing in the early period due to war and fire. During 960–1500 CE, drought and flood records existed only for some periods with prosperous dynasties. For instance, records were relatively complete, and the average missing rate was less than 20% in areas from North China to the Jianghuai area of the *North-Song* Dynasty (960–1100 CE), the lower reaches of the Yangtze River of the mid-*South-Song* Dynasty (1101–1200 CE), and areas from North China to the middle and lower reaches of the Yangtze River of the mid-*Ming* Dynasty (1401–1500 CE). However, the rate of missing data was even greater than 90% in Northwest China, Southwest China, and South China during 960–1500 CE (Fig. 2b,g). However, there are no missing data since 1501 AD (Fig. 2a). To date, this dataset has been widely used to reconstruct gridded precipitation/hydroclimate dataset in EC during the past 500 years^26–28^, as well as to analyse of extreme hydroclimate variations over the last millennium^30,44^.

A total of 5292 tree-ring chronologies in the NH are derived from World Data Service (WDS) for Paleoclimatology Data (https://www.ncdc.noaa.gov) (Fig. 1), including 4513 (85.28% of the total samples) tree-ring width chronologies, 770 (14.55%) tree-ring density chronologies, and 9 (0.17%) tree-ring oxygen isotope chronologies, which are mainly located in Asia, Europe, and North America. In detail, tree-ring sample sites in Asia (excluding western Russia) are distributed in the Mongolian Plateau, Tianshan Mountain, Qilian Mountain, south and southeast of the Tibetan Plateau, Japan, and the China-Indochina Peninsula. The 1857 tree-ring chronologies in Asia consisted of 1488 (80.13% of the total samples in Asia) tree-ring width chronologies, 770 (19.39%) tree-ring density chronologies, and 9 (0.48%) tree-ring oxygen isotope chronologies. With the exception of Belarus and Ukraine, the tree-ring sample sites cover almost the entire European region, with a total of 1846 chronologies which consist of 1436 (77.79% of the total samples in Europe) tree-ring width chronologies and 410 (22.21%) tree-ring density chronologies. The North American tree-ring samples cover the contiguous United States (excluding Alaska and Hawaii), Mexico, and most of Canada, with a total of 1589 tree-ring width chronologies. The lengths of tree-ring chronologies in the NH vary, with the majority ending after the 1970s. The samples for which the last year of chronology occurred in the 1970s, 1980s, 1990s and 2000s accounted for 16.67%, 18.99%, 37.62% and 20.39% of the total, respectively. Moreover, the numbers of chronologies in the above four periods whose first years are earlier than 1500 CE are 46, 119, 151, and 144, respectively. This indicates that tree-ring chronologies longer than 500 years mostly end after the 1980s. In terms of eastern China, to supply the data missing due to a lack of DFGs before 1500 CE, tree-ring chronologies from the 1980s were selected to establish a regression model and reconstruct the gridded D/F grades dataset in EC.

The speleothem chronology in eastern China is also derived from the WDS for Paleoclimatology Data (https://www.ncdc.noaa.gov) (Fig. 1). Speleothem chronology has decadal to multidecadal resolution using the U-Th dating technique. In this study, one speleothem chronology, located in Dayu Cave (106.3°E, 33.13°N) was selected. It spanned 1249 CE to 1983 CE, with a temporal resolution of approximately 3–4 years and a correlation coefficient of −0.22 with local summer precipitation.

### Instrumental precipitation dataset

The monthly instrumental precipitation dataset GPCC V7 with 0.5- by 0.5-degree spatial resolution from 1891 to 2015 was used in this study, which was provided by the Global Precipitation Climatology Centre^45^. This set of data is developed through SPHEREMAP interpolation of 85,000 stations, which are derived from ground site-based measurements from 158 countries and 31 regional agencies, and daily surface weather observations are from the Global Telecommunications System (GTS) of the World Meteorological Organization (WMO). Moreover, interpolation methods take into account the influence of station relocation and instrumental replacement. Because of large uncertainties in the interpolation results caused by the heterogenous spatial distribution of global meteorological stations in the early 20^th^ century, we utilized monthly precipitation during 1921–1980 as calibration data to construct the regression equations.

### Simulation dataset

This study used climate modeling data of full forcing experiments, including geopotential height, u-wind, v-wind and vertical wind, which were derived from CESM-LME. And, simulations span the years 850–2005 providing 13 simulated cases of ensemble of last millennium. CESM-LME is a fully coupled general circulation model linking atmosphere, ocean, land, and sea ice components of the climate system developed at the National Center for Atmospheric Research (NCAR). This model uses the version 1.1 of CESM with the Community Atmosphere Model version 5 (CESM1-CAM5) run at ~2-degree resolution in the atmosphere and land components and ~1 degree resolution in the ocean and sea ice components. Characteristics of each climate forcing are listed in Otto-Bliesner *et al*.^46^. The full forcing experiments employ real-time, dynamic external forcing consisting of volcanic aerosols, solar irradiation, GHGs, and LUCC^47^. Thereby, full forcing experiments represent climate changes driven together by both external forcing and internal variability.

## Data Records

The 2.5-degree annual resolution D/F grades dataset for EC in the period of 960–2000 CE in this study is hosted at the World Data Center for Climate (10.26050/WDCC/HydrocGDForEChinaTheLastMillV10) with three sets of files (Table 1): (a) reconstructed D/F grades dataset based on proxy data with 2.5-degree spatial resolution from 960–2000 CE, including 1 file named named Hydro_EC.mat; (b) validation parameters of the calibration equations for 960–1500 CE, such as the adjusted R2, predicted R2, RE and CE, are also included in 1 file named Val_Par.mat; (c) sampling error estimates of interpolated dataset for 1500–2000 CE, in another 1 file named SEE.mat^48^.

**Table 1:** Detailed information about the reconstructed hydroclimate and validation dataset.

	Metrics	Spatial Resolution	Temporal Resolution	Coverage Period
Hydro_EC	drought/flood grades	2.5° × 2.5°	annual	960–2000 CE
Val_Par	adjusted R^2^, predicted *R*^2^, RE and CE	2.5° × 2.5°	—	—
SEE	sampling error	2.5° × 2.5°	—	—

## Technical Validation

### Error estimation of the interpolated result for 1500–2000

**Fig. 3:**
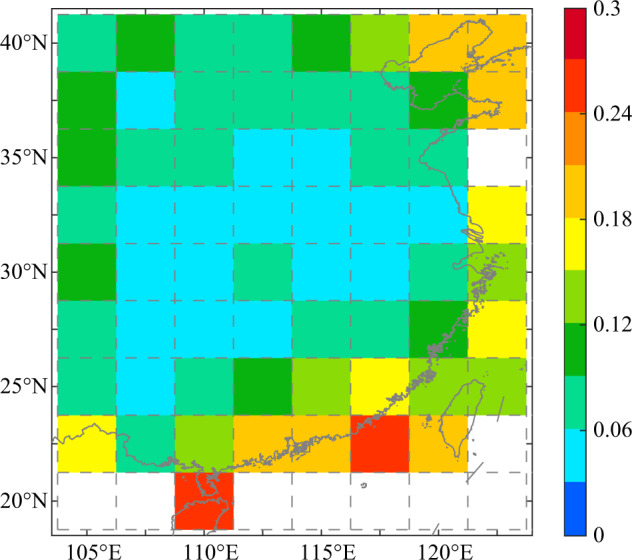
Sampling errors estimate for hydroclimate interpolation results from 1500 to 2000.

Based on the ADW method, DFG data in EC during 1500–2000 were interpolated to obtain a gridded EC D/F grades dataset. Both the estimation of sampling error and correlation analysis were investigated to verify the reliability of the gridded D/F grades dataset. Figure 3a shows the spatial pattern of the sampling error estimates. The sampling error estimates were 0.04–0.27. The middle and lower reaches of the Yellow River valley have the lowest error of less than 0.06, due to the increased sample size retrieved in each grid. With the decrease in the sample size, the sampling error increases obviously for other regions, i.e., reaching 0.06–0.12 in North China Plain, northwestern China, and portions of the southeast coastal region and 0.18–0.27 for northeastern China and portions of southern China. Although the maximum value of the sampling error estimate was 0.27, it was still less than 0.5. Compared to DFGs data, the systematic errors generated by the interpolation process did not result in dryness/wetness type changes in the gridded hydroclimate.

### Correlation analysis

**Fig. 4:**
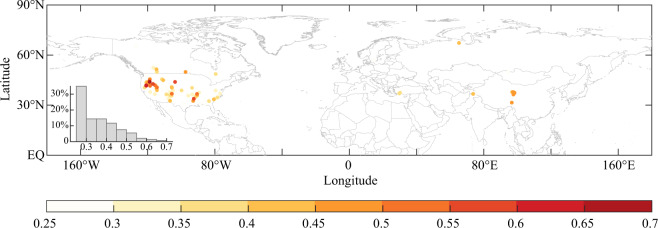
Distribution of tree-ring chronologies with a significant (*p* < 0.05) positive correlation with local summer precipitation and distribution probability of correlation coefficients.

Figure 4 shows that tree-ring width chronologies revealing local summer precipitation variations were mainly in the United States (US) and northwestern China. In the US, the correlation between tree-ring chronologies and summer precipitation gradually weakened from northwest to southeast. The tree-ring width growth in the northwest portions was more significantly affected by precipitation variations, and the average correlation coefficients (*r*) in the northern Rocky Mountains reached over 0.6, while the average *r* in the Great Plain and southeastern coastal region was 0.35. In northwestern China, the average *r* was almost 0.45, indicating that local precipitation also had a significant impact on the radial growth of tree-ring width. Overall, the tree-ring chronologies with *r* > 0.5 accounted for approximately 22% of the total TRCs, which were distributed in the US and northwestern China. The tree-ring chronologies with *r* > 0.6 accounted for approximately ~5%, and were distributed only in the northwestern US.

### Validation and selection of climate model

**Fig. 5:**
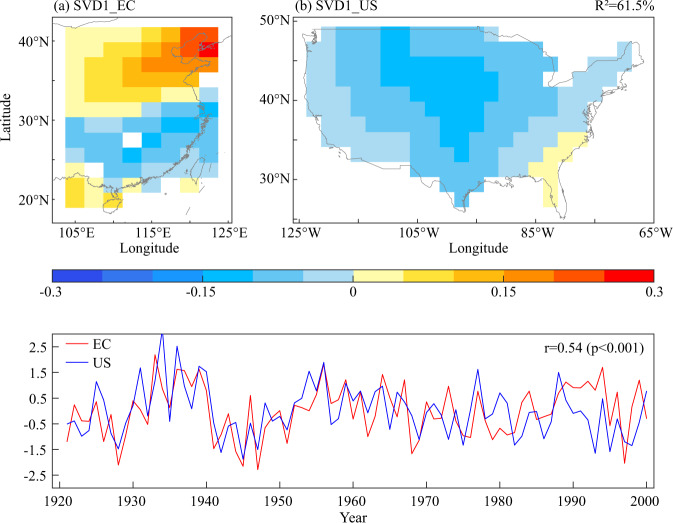
Spatial pattern for the first leading SVD mode between eastern China (a) and the United States (b) in 1921–2000 based on GPCC observational precipitation.

This study compared the spatial pattern of teleconnection of observation data and simulation data in order to select the optimal climate model cases from CESM-LME using the SVD approach. The SVD analysis of GPCC precipitation variations during 1921–2000 was used to diagnose the potential teleconnection pattern between EC and the US, as shown in Fig. 5. The *R*^2^ values of the first mode was 61.5%, which indicated that the first mode was sufficient to reveal the main characteristics of covarying precipitation in EC and the US. The first mode (SVD1) in Fig. 5 shows that the spatial pattern of EC was dominated by inconsistent precipitation variations with a south-north dipole mode, a positive centre was in northern China, and a negative centre was in southern China. With the exception of the southeastern US (mainly Florida State), the US demonstrated consistent precipitation variations across the most regions, which were centred on the middle portion of the Great Plains. The covarying precipitation pattern in EC and the US was as follow: “southern China-US and northern China” pattern. The spatial correlation pattern between TRCs in the United States/northwestern China and gridded hydroclimate in EC were investigated. Compared with the SVD1 mode, TRCs capable of indicating teleconnection patterns were selected. All chronologies that showed consistency with the SVD1 spatial pattern were chosen by human identification. A total of 18 tree-ring chronologies in the US fit the criteria (figure not shown). 13 tree-ring chronologies were distributed in the southwest while five TRCs were distributed in the northeast. The abovementioned findings indicated that TRCs reflecting the SVD1 mode were mainly located in the southwestern United States. Notably, some chronologies in the United States were related to the hydroclimate in portions of EC, which may be caused by tree rings explaining parts of precipitation variations.

**Fig. 6:**
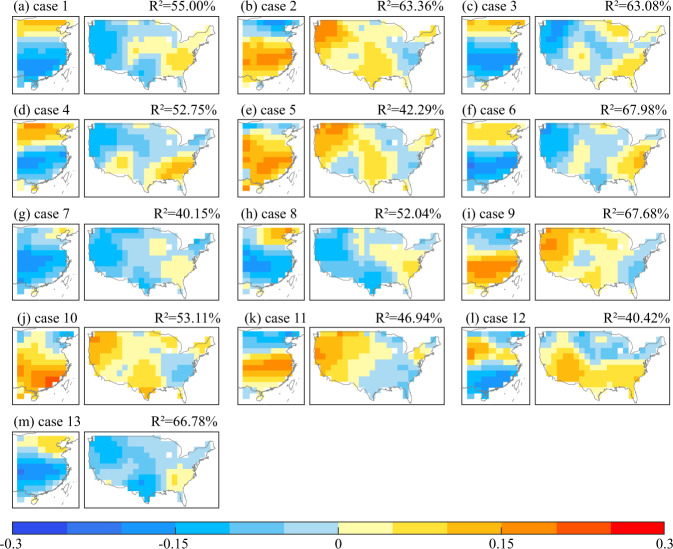
Spatial pattern for the first leading SVD mode between eastern China (a) and the United States (b) in 1921–2000 based on 13 cases of full forcing experiment in CESM-LME.

The SVD analysis of a total of 13 CESM-LME simulation cases precipitation variations during 1921–2000 was used to diagnose the potential teleconnection pattern between EC and the US, as shown in Fig. 6. The ranges of *R*^2^ values of the first mode were 40.15%–61.5%. Additionally, we found that a total of 6 CESM-LME simulation cases of case 2, case 3, case 4, case 6, case 9, case 13 could reproduce the spatial teleconnection pattern by comparing with the simulation results and observation results to spatial pattern of teleconnection between EC and the United Staten. The covarying precipitation pattern in EC and the US in these six cases were as follow: “southern China-US and northern China” pattern. The *R*^2^ values of the first mode in the six cases in CESM-LME were at least 52.75%, which indicated that the first modes in the six cases in CESM-LME were sufficient to reveal the main characteristics of covarying precipitation in EC and the US. And in the following, we used the MME of the six cases in CESM-LME to diagnose the potential causes of teleconnection mode between EC and the United States.

### Diagnosis of the teleconnection mode of precipitation variations

**Fig. 7:**
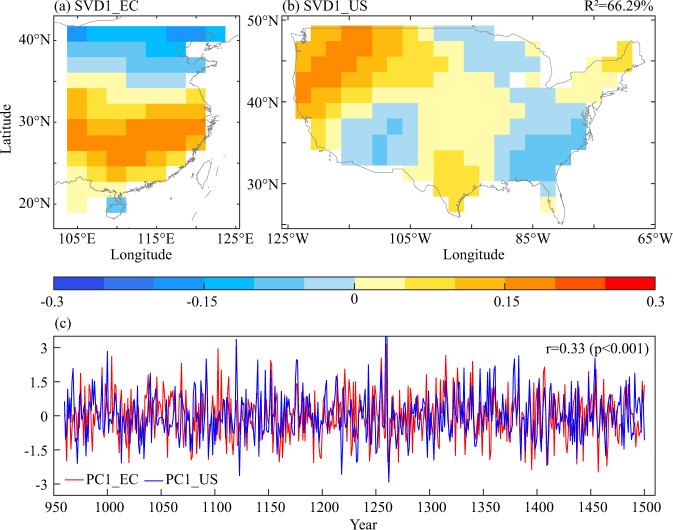
Spatial pattern for the first leading SVD mode between eastern China (a) and the United States (b) and time series (c) in 960–1500 based on CESM-LME.

The SVD analysis of MME of CESM-LME precipitation variations during 960–1500 was used to diagnose the potential teleconnection pattern between EC and the US, as shown in Fig. 7. The *R*^2^ values of the first mode was 66.29%. which indicated that the first mode was sufficient to reveal the main characteristics of covarying precipitation in EC and the US. The first mode (SVD1) in Fig. 7 showed that the spatial pattern of EC was dominated by inconsistent precipitation variations with a south-north dipole mode, a positive centre was in southern China, and a negative centre was in northern China. The US demonstrated inconsistent precipitation variation patterns, which were negative in the southwestern and the southeastern US, and positive in the northwestern US and Great Plain. Additionally, the time series correlation of between EC and the United States was 0.33 (*p* < 0.001), suggesting that the spatial teleconnection between the two regions was significant during 960–1500.

**Fig. 8:**
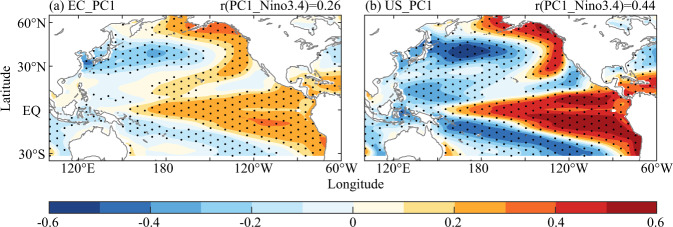
Spatial pattern of the regression coefficient of the time series of SVD1 and EC (**a**) and the United States (**b**) and the sea surface temperature anomaly respectively (The black dots denote significance test at a 0.1 level; the top right corners represent the correlation coefficient of PC1 and sea surface temperature anomaly of Niño3.4).

Figure 8 showed that spatial pattern of the regression coefficient of the time series of SVD1 of EC and the United States and the sea surface temperature (SST) anomaly. In the Equatorial East Central Pacific, we discovered significant El Niño mode, and the correlation coefficients between PC1 in the EC and the United States and SST anomalies of Niño3.4 were 0.26 (*p* < 0.05) and 0.44 (*p* < 0.05) from 960 to 1500, respectively. Meanwhile, there were significant warm phase PDO mode in the North Pacific. This suggested that the spatial pattern of hydroclimate teleconnection between EC and the United States may be triggered by the occurrence of El Niño in the context of warm phase PDO. Importantly, El Niño occurred in the context of warm phase PDO.

**Fig. 9:**
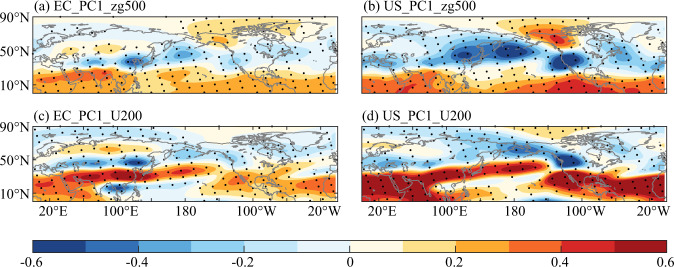
Spatial pattern of the regression coefficient of the time series of SVD1 of EC and the United States with the geopotential height anomaly of the 500 hPa pressure level and zonal wind anomaly of the 200 hPa pressure level respectively (The black dots denote significance test at a 0.1 level).

The geopotential height significantly decreased at mid-latitudes for both PC1 in EC and PC1 in the United States, according to the spatial pattern of the regression coefficient of the time series of SVD1 of EC and the United States with the geopotential height anomaly of the 500 hPa pressure level (Fig. 9a,b). In detail, significant negative centres included EC, the United States, and the North Pacific, which enhanced the development of eastern Rossby waves. The geopotential height patterns at mid-latitudes corresponded to the teleconnection patterns of precipitation. Furthermore, the circumpolar Arctic and low latitudes displayed significant positive. From the spatial pattern of the regression coefficients of the 200hPa zonal wind (Fig. 9c,d), there was significant wind shear in EC and the boreal, and similarly in the United States and the boreal. There were notable significant positive anomalies in eastern China and the United States, and significant negative anomalies in the boreal. The westerly pattern was matched with the teleconnection pattern of precipitation in above-mentioned regions. Additionally, From the spatial pattern of the regression coefficients of the 850hPa wind (Fig. 9c,d), in contrast to the southern China, where there were some southerlies that supported water vapor transfer, the dominant wind in northern China was northerly, which was not favourable to water vapor transport and resulted in decreasing in precipitation. While the majority of the western United States experienced southwesterlies, which were constantly carried by water vapor from the Pacific and increased precipitation. The perspective of large-scale horizontal circulation suggested that the occurrence of El Niño in the context of warm phase PDO was likely to promote the sinking of mid-latitude geopotential height and the enhancement of wind shear in the westerly zone, and the transport of water vapor from the Pacific Ocean led to increased precipitation in southern China and parts of the United States.

**Fig. 10:**
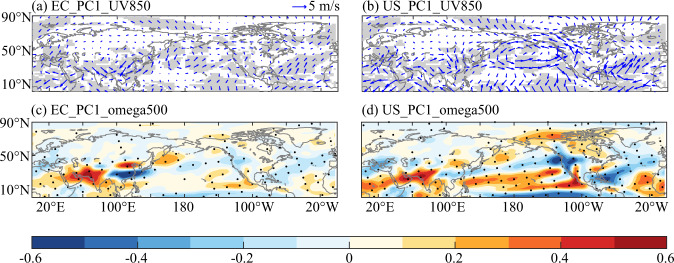
Spatial pattern of the regression coefficient of the time series of SVD1 of EC and the United States with the wind anomaly of the 850 hPa pressure level and vertical wind anomaly of the 500 hPa pressure level respectively (The grey shadings and the black dots denote significance test at a 0.1 level; sigma < 0 denotes upward and sigma > 0 denotes downward).

From the spatial pattern of the regression coefficients of the 500hPa vertical wind (Fig. 10c,d), northern China was mainly downward movement, accompanied by scarcity of horizontal water vapor conditions, which would be unfavourable for precipitation. It was opposite of the northern China. There was upward movement in southern China and accompanied by a large amount of horizontal water vapor transport from the Pacific Ocean. When the lifting condensation height was reached, horizontal and vertical circulation combined to promote precipitation. And, there was similar causes in the United States. The southwestern part of the United States was characterized by downdrafts, while most other regions were characterized by updrafts. For instance, precipitation will increase over the northwestern United States due to significant water vapor transport and upward movements. The spatial pattern of vertical circulation was also more consistent with the above-mentioned spatial pattern of teleconnection of precipitation. According to the vertical circulation perspective, the vertical circulation would further encourage/suppress the occurrence of precipitation in different regions under the conditions of horizontal water vapor transport.

Importantly, the hydroclimatic teleconnection patterns in the midlatitudes of the Northern Hemisphere are regulated by the Circumglobal Teleconnection (CGT)^49–51^. In detail, the CGT mode alters the development of the eastward Rossby wave train in two ways. The first cause is the direct effect of El Niño. A strong El Niño may lead to equatorial tropospheric temperature warming and then excite a zonal seesaw structure between the equator and temperate zone by the enhancement of the Hadley circulation. This may largely stimulate zonally symmetric wave trains^52,53^. The second cause is the indirect effects of El Niño. A strong El Niño may lead to negative summer monsoon precipitation in India. Less precipitation may decrease sensible heat and increase latent heat through the local feedback of land–air interactions. As the strongest continental heat source at low latitudes, diabatic heating of local air may lead to a baroclinic structure over the troposphere. Baroclinic instability would excite successive downstream cells along the waveguide through Rossby waves. Ding *et al*. (2005, 2011)^49,50^ and Hao *et al*. (2021)^51^ suggested that the abovementioned two processes may stimulate meridional disturbance of eastward Rossby wave trains at midlatitudes and then change the intensities and locations of the troughs and ridges and even water vapor transport over the NH (i.e., EC and the United States) from modern and historical climate perspectives, respectively. Therefore, the hydroclimatic teleconnection pattern between selected tree-ring sites (i.e., the United States and northwestern China, etc.) and EC were identified. Through the identified hydroclimatic teleconnection pattern, the spatial correlation relationship was constructed between TRCs in the United States/northwestern China and gridded hydroclimate in EC. The tree-ring chronologies in the United States and northwestern China that were chosen were highly correlated with EC hydroclimate change on a large scale and matched with the teleconnection spatial pattern of hydroclimate. To some extent, such TRC variations were closely related to hydroclimate variations in EC.

Many spatial correlation patterns between tree-ring chronologies and the hydroclimate in EC showed consistencies with identified spatial patterns of the first two SVDs. Thus, teleconnection patterns for precipitation variations between EC and the US were used for investigating the potential linkages between US tree-ring width variations and EC hydroclimate, and for hydroclimate reconstructions. Compared with the SVD1 mode, TRCs capable of indicating teleconnection patterns were selected. All chronologies that showed consistency with the SVD1 spatial pattern were chosen by human identification. A total of 18 tree-ring chronologies in the US fit the criteria, as illustrated in Fig. S1. 13 tree-ring chronologies were distributed in the southwest while five tree-ring chronologies were distributed in the northeast. The abovementioned findings indicated that TRCs reflecting the SVD1 mode were mainly located in the southwestern United States. Notably, some chronologies in the United States were related to the hydroclimate in portions of EC, which may be caused by tree rings explaining parts of precipitation variations.

### Validation of the calibration equation for 960–1500

Based on the identified teleconnection modes, the gridded EC hydroclimate was reconstructed from 960 to 1500. However, due to the length limitation of tree-ring chronologies or missing DFGs values, the lengths of the reconstruction for three grids were shorter than that for the last millennium, i.e., 105°E and 25°N, 110°E and 25°N, and 120°E and 40°N, with missing rates of 23.63%, 51.10%, and 8.36%, respectively. Figure 7a,b show the mean ${R}_{a}^{2}$ and ${R}_{pr}^{2}$ for the calibration equation of 1921–1980, respectively. The ${R}_{a}^{2}$ values were 27.49%–81.78%, and the average value was 59.97%. The range of ${R}_{pr}^{2}$ values were 25.19%–76.48%, and the average value was 54.67%. The significant explained variance was extended southward from the North China Plain to the middle and lower reaches of the Yangtze River, reaching more than 0.6, due to tree-ring chronologies that were significantly related to the hydroclimate of these regions. In some regions, such as southwestern China and portions of South China, the *R*^2^ values declined to 0.25–0.5. As shown in Fig. 3, the sampling error estimates in these regions were relatively higher than those in other regions. As a result, this may lead to the inability to find sufficient correlated chronologies with EC hydroclimate. Although the explained variance in portions of southwestern China was 0.25, such reconstructed results were adequate and plausible from the tree-ring climatology perspective.

**Fig. 11:**
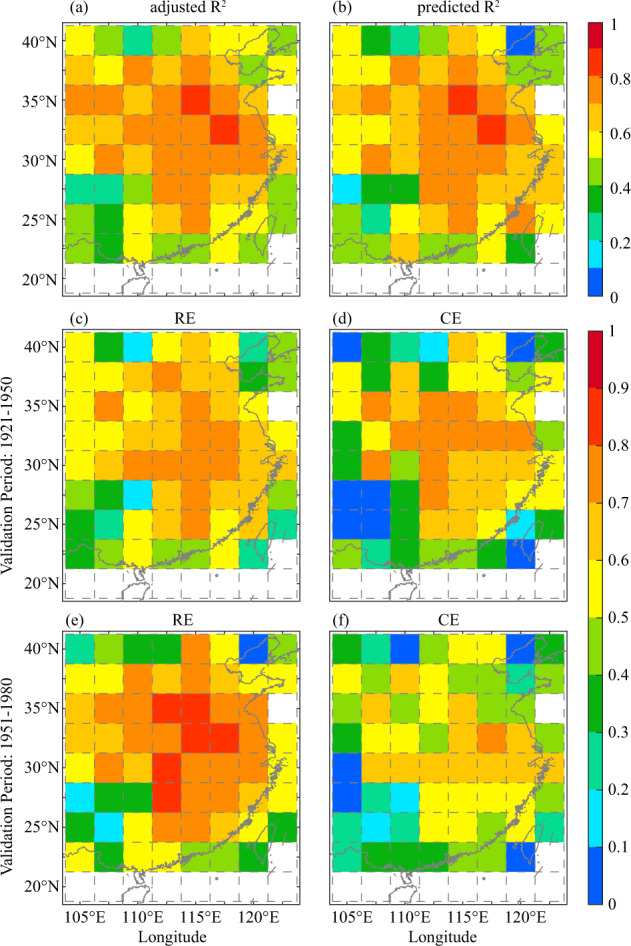
Mean adjusted *R*^2^ (**a**), mean predicted *R*^2^ (**a**), and RE and CE for the cross calibration and validation in the periods of 1921–1950 and 1951–1980 respectively (**c**–**f**) for hydroclimate reconstruction from 960 to 1500.

In addition, some statistical parameters of the calibration equations, including mean RE and CE for the cross calibration and validation, are shown in Fig. 7c–f. The calibration period was 1951–1980 for RE and CE, their validation period was 1921–1950 (Fig. 11c,d), and vice versa in Fig. 7e,f. All RE and CE values were greater than zero over the calibration and validation periods, indicating that the calibration equations were stable and reliable and could be utilized to reconstruct EC hydroclimate. However, there were some spatial differences in RE and CE. Regardless of whether the validation period was 1921–1950, or 1950–1980, large values of RE and CE were displayed in North China Plain and the middle and lower reaches of the Yangtze River, and the mean RE and CE reached more than 0.6. In southwestern China and northeastern China, RE and CE for the cross-calibration and validation were low, and the average reached 0.2–0.4. These results indicated that the calibration equations in the North China Plain and the middle and lower reaches of the Yangtze River were more stable and that the effects of the reconstructed results were better than those in other regions. The abovementioned findings suggested that the average explained variance of the reconstructed hydroclimate in most EC was more than 50%, and a reconstructed gridded D/F grades dataset was available.

### Difference of spatial pattern under different warm/cold climate background

**Fig. 12:**
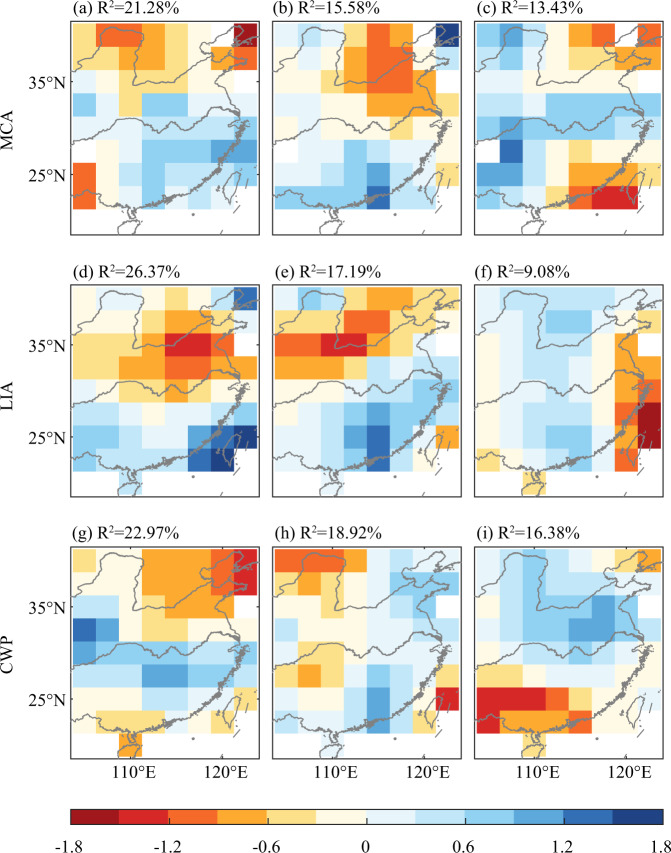
Spatial pattern of first three modes of EOF for hydroclimate in the period of Medieval Climate Anomaly (**a**–**c**), Little Ice Age (**d**–**f**), and Current Warm Period (**g**–**i**).

Using the reconstructed D/F grades dataset in this study, we analysed the dominant spatial pattern of EOFs in EC in the context of the Medieval Climatic Anomaly (MCA), Little Ice Age (LIA), and Modern Warm Period (MWP). Figure 12 illustrated that the *R*^2^ of the first three EOF spatial patterns in MCA were 21.28%, 15.58% and 13.43%, respectively, adding up to 50.29%. The R^2^ of the first three EOF spatial patterns in LIA were 26.37%, 17.19% and 9.08%, respectively, adding up to 52.64%. The R^2^ of the first three EOF spatial patterns in MWP were 22.97%, 18.92% and 16.38, respectively, adding up to 58.27%. The first three EOF modes’ all combined R^2^ values in the three cold and warm climate background exceeded 50%, which suggested that these could be used to roughly represent the dominating spatial patterns in the corresponding period. During the MCA, the first mode was “North China-the middle and lower reaches of Yangtze River to South China” in dipole pattern; the second mode was “North China to the middle and lower reaches of Yangtze River–South China” in dipole pattern; the third mode was “North China–middle and lower reaches of Yangtze River–South China” in tripolar pattern. The EOF1 of the LIA was similar with the EOF2 spatial pattern of the MCA, and the EOF2 of the LIA was similar with the EOF1 spatial pattern of the MCA. This suggested that both the MCA and LIA had similar dominant patterns, but the dominant patterns were ranked differently, which may have been affected by different warm and cold climate background. The third mode in LIA was “central and western–coastal” in dipole pattern. The EOF1 of the MWP was basically similar to that of the MCA, indicating that the dominant spatial pattern in the warm climate background was “North China–the middle and lower reaches of Yangtze River to South China”. The EOF2 of the MWP was an “east–west” antiphase spatial pattern. The EOF3 was like the EOF2 of the MCA, i.e., the “North China to Middle and Lower Yangtze River - South China” antiphase spatial pattern.

Above-mentioned findings suggested that there are some differences in the dominant spatial patterns of interdecadal hydroclimate variations in EC under different cold and warm climate background in the last millennium. Meanwhile, climate backgrounds with various rates of warming could result in variations in spatial patterns even when they share the same warming situation.

## Usage Notes

This data is intended for use in comparing with spatial hydroclimatic pattern among Medieval Climate Anomaly, Little Ice Age and Current Warm Period, and investigating the characteristics of hydroclimate extremes, as well as the relationship with climate modes, such as ENSO, PDO, AMO. The last millennium D/F grades dataset in eastern China with 2.5° resolution from 960 to 2000, including 1 file named Hydro_EC.mat. And for 960–1500 CE, the validation parameters of the calibration equations, such as the adjusted *R*^2^, predicted *R*^2^, RE and CE, are also included in 1 file named Val_Par.mat; for 1500–2000 CE, the sampling error estimates of interpolated dataset are in another 1 file named SEE.mat. 10.26050/WDCC/HydrocGDForEChinaTheLastMillV10.

## Supplementary information

Supplementary Information

## Data Availability

All calculations of hydroclimate reconstruction for eastern China are based on the MATLAB language and are available at GitHub: https://github.com/baimengxin2016/Hydro_EC. Any updates will also be published on GitHub.
