# Supplementary material for: A 2.5° × 2.5° gridded drought/flood grades dataset for eastern China during the last millennium
Source: Sci Data. 2023 Apr 11;10:202. doi: 10.1038/s41597-023-02110-5 (PMC10090067; doi:10.1038/s41597-023-02110-5)
Supplement: Supplementary file 1 — Supplementary Information [file 41597_2023_2110_MOESM1_ESM.docx]

**Supporting Information for “A 2.5°×2.5° gridded drought/flood grades dataset for eastern China during the last millennium”**

Zhixin Hao^1,2*^, Jingyun Zheng^1,2^, Quansheng Ge^1^, Mengxin Bai^3,1*^

1 Key Laboratory of Land Surface Pattern and Simulation, Institute of Geographic Sciences and Natural Resources Research, Chinese Academy of Sciences, Beijing, 100101, China

2 University of Chinese Academy of Sciences, Beijing, 100049, China

3 Beijing Municipal Climate Center, Beijing Meteorological Bureau, Beijing, 100089, China

Corresponding author1: Prof. Zhixin Hao

Address: 11A Datun Road, Chaoyang District, Beijing, China

E-mail: [haozx@igsnrr.ac.cn](mailto:haozx@igsnrr.ac.cn)

Corresponding author2: Dr. Mengxin Bai

Address: 44 Zizhuyuan Road, Haidian District, Beijing, China

E-mail: [bmengxin@163.com](mailto:bmengxin@163.com)


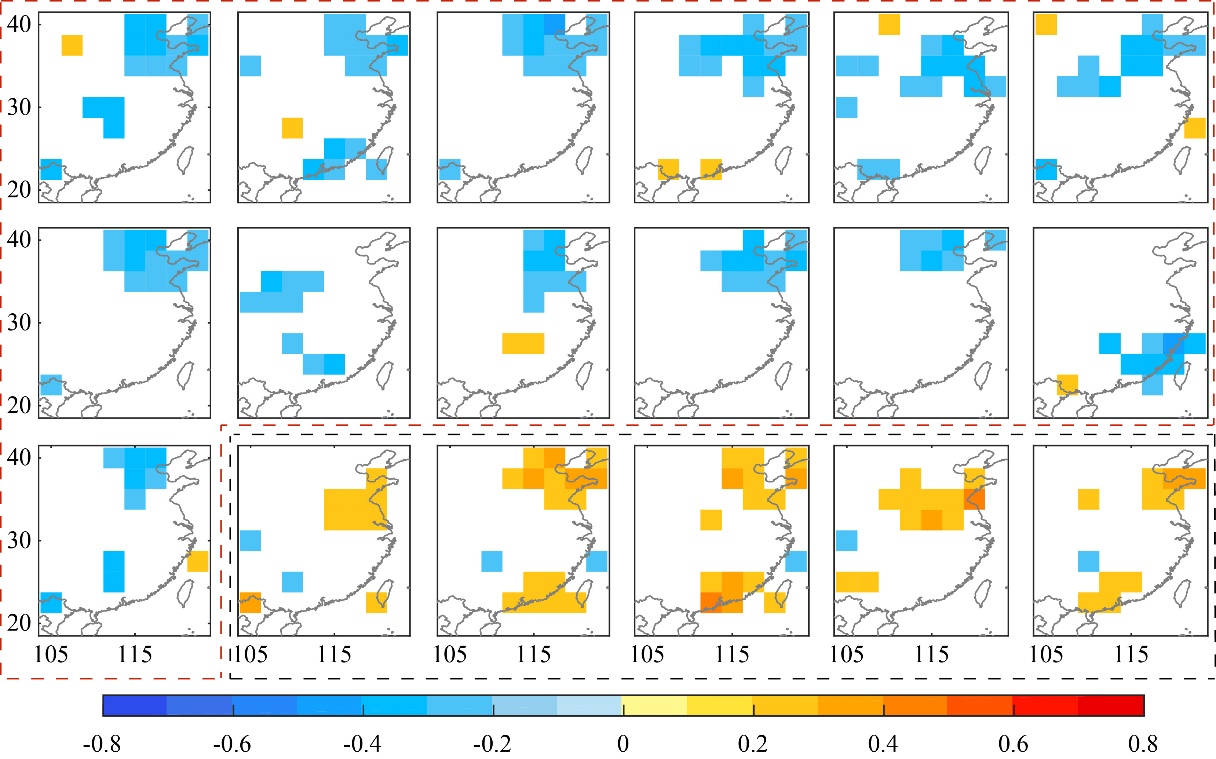


**Figure S1** Spatial patterns of correlations matching with SVD 1 between tree-ring chronologies in the United States and EC hydroclimate (Filling values denote passing the 95% significance test; within red dashed line denotes these tree-ring sites are in the southwestern United States; within black dashed line denotes these tree-ring sites are in the northeastern United States)

**Table S1** Calibrated models and $R_{a}^{2}$ of hydroclimate reconstruction for different periods in the grid (115°E,35°N)

| **reconstructed period** | **regression model** | $\boldsymbol{R}_{\boldsymbol{a}}^{\boldsymbol{2}}$ |
| --- | --- | --- |
| 960-961 | y=2.97+0.13DFG1+0.08DFG3+0.26DFG4+0.09DFG6+0.51DFG7  +0.20DFG8+0.11DFG10+0.20DFG14-0.12tree1+0.05tree2+0.07tree4 | 0.83 |
| 962; 1010; 1016; 1027; 1048;1077; 1080; 1113-1114; 1120-1121; 1125-1126; 1297;1310-1311; 1329-1331; 1431; 1445-1447; 1462 | y=2.97+0.07DFG1+0.31DFG4+0.16DFG5+0.04DFG6  +0.78DFG7+0.06DFG9+0.10DFG13+0.13DFG16 | 0.85 |
| 963-966; 973-974; 987; 996; 998-1012; 1026;1035;1043; 1046; 1051; 1053-1055; 1083; 1095; 1122; 1157; 1177; 1192; 1211-1213; 1264; 1271; 1278; 1285-1286; 1295-1296; 1303-1306; 1312; 1321; 1323; 1332; 1342; 1359-1360; 1372-1373; 1375; 1403-1404; 1408-1410; 1416-1417; 1425; 1428; 1436; 1440-1444; 1450-1451; 1455-1456; 1459; 1470; | y=2.95+0.08DFG1+0.29DFG4+0.16DFG5+0.05DFG6+0.50DFG7  +0.24DFG8+0.08DFG13+0.15DFG14+0.14DFG16-0.06tree1+0.03tree2 | 0.85 |
| 967; 1092; | y=2.98+0.27DFG4+0.17DFG5+0.04DFG6+0.52DFG7+0.24DFG8  +0.16DFG14+0.17DFG16-0.06tree1+0.07tree2 | 0.84 |
| 968 | y=2.97+0.10DFG1+0.26DFG4+0.12DFG5+0.06DFG6+0.53DFG7+0.19DFG8  +0.09DFG10+0.02DFG12+0.08DFG13+0.17DFG14-0.06tree1+0.03tree2 | 0.84 |
| 969-970; 972; 992; 1013; 1078; 1082; 1091; 1176; 1191; 1206; 1287-1288; 1309; 1324-1326; 1343; 1411-1413; 1435; 1437-1439; 1448-1449; 1453-1454; 1457-1458 | y=2.94+0.08DFG1+0.29DFG4+0.16DFG5+0.05DFG6+0.50DFG7+0.24DFG8  +0.08DFG13+0.15DFG14+0.14DFG16-0.06tree1+0.03tree2 | 0.85 |
| 971 | y=2.93+0.27DFG4+0.15DFG5+0.04DFG6+0.52DFG7+0.21DFG8+0.05DFG12  +0.06DFG13+0.16DFG14+0.16DFG16-0.05tree1+0.01tree1 | 0.85 |
| 975; 1023; 1098; 1101; 1186; 1215; 1260-1261; 1279 | y=2.94+0.09DFG1+0.28DFG4+0.15DFG5+0.05DFG6+0.26DFG8  +0.17DFG14+0.15DFG16-0.05tree1+0.03tree1 | 0.85 |
| 976 | y=2.96+0.29DFG4+0.76DFG7+0.18DFG8+0.03DFG11  +0.14DFG13-0.14tree1+0.04tree3+0.11tree4 | 0.82 |
| 977; 1073; 1204; 1208; 1262-1263; 1341; 1346; 1386-1387; 1392; 1405-1406 | y=2.95+0.10DFG1+0.11DFG3+0.29DFG4+0.07DFG6+0.49DFG7+0.24DFG8  +0.09DFG13+0.16DFG14+0.13DFG16-0.13tree1+0.03tree2+0.07tree3+0.05tree4 | 0.84 |
| 978; 986; 1032; 1123; 1168-1169; 1180; 1234; 1239-1240; 1380-1381; 1468 | y=2.97+0.10DFG1+0.09DFG3+0.29DFG4+0.09DFG6+0.46DFG7  +0.25DFG8+0.19DFG14+0.14DFG16-0.09tree1+0.05tree2+0.05tree4 | 0.84 |
| 979-980; 1015; 1040; 1060; 1129; 1218; 1223; 1225-1226; 1233; 1397; 1398 | y=2.97+0.33DFG4+0.11DFG6+0.48DFG7+0.22DFG8+0.19DFG14  +0.15DFG16-0.07DFG17+0.02DFG18+0.02DFG20 | 0.83 |
| 981; 1047; 1216-1217 | y=2.97+0.27DFG4+0.17DFG5+0.04DFG6+0.52DFG7+0.24DFG8  +0.16DFG14+0.17DFG16-0.06DFG17+0.01DFG18 | 0.84 |
| 984; 991; 1001; 1004-1006; 1045; 1052; 1068; 1072; 1102-1103; 1108-1109; 1127-1128; 1161; 1167; 1200; 1266-1268; 1292-1293; 1313; 1322; 1335; 1339-1340; 1345; 1376-1377; 1390-1391; 1393-1394; 1402; 1424; 1466-1467 | y=2.97+0.10DFG1+0.11DFG3+0.29DFG4+0.07DFG6+0.49DFG7+0.24DFG8  +0.09DFG13+0.16DFG14+0.13DFG16-0.13DFG17+0.03DFG18+0.07DFG19+0.05DFG20 | 0.84 |
| 985; 990; 1107; 1349; 1389 | y=2.98+0.08DFG1+0.11DFG3+0.26DFG4+0.10DFG6+0.83DFG7  +0.13DFG13-0.14DFG17+0.10DFG19 | 0.83 |
| 988; 1050; 1067; 1265; 1280-1281; 1289; 1350; 1358 | y=2.96+0.10DFG1+0.26DFG4+0.14DFG5+0.57DFG7+0.29DFG8-0.01DFG12  +0.10DFG13+0.13DFG14-0.08DFG17-0.01DFG18+0.04DFG19+0.07DFG20 | 0.84 |
| 989; 1042; 1277; 1282; 1348; 1365-1367 | y=2.96+0.10DFG1+0.26DFG4+0.14DFG5+0.57DFG7+0.29DFG8-0.01DFG12  +0.10DFG13+0.13DFG14-0.08DFG17-0.01DFG18+0.04DFG19+0.07DFG20 | 0.84 |
| 994-995; 1002; 1058 | y=2.97+0.10DFG1+0.09DFG3+0.29DFG4+0.09DFG6+0.46DFG7+0.25DFG8  +0.19DFG14+0.14DFG16-0.09DFG17+0.05DFG18+0.05DFG20 | 0.84 |
| 997; 1044; 1207 | y=2.97+0.28DFG4+0.15DFG5+0.04DFG6+0.52DFG7+0.21DFG8+0.05DFG12  +0.06DFG13+0.16DFG14+0.16DFG16-0.05DFG17+0.01DFG18 | 0.85 |
| 1003; 1029; 1061-1062; 1079; 1093; 1209-1210; 1270; 1301-1302; 1314-1315; 1319 | y=2.96+0.10DFG1+0.11DFG3+0.29DFG4+0.07DFG6+0.49DFG7+0.24DFG8+0.09DFG13  +0.16DFG14+0.13DFG16-0.13DFG17+0.03DFG18+0.07DFG19+0.05DFG20 | 0.84 |
| 1014; 1181; 1188; 1189 | y=2.97+0.1DFG1+0.09DFG3+0.29DFG4+0.09DFG6+0.46DFG7  +0.25DFG8+0.19DFG14+0.14tree1-0.09tree2+0.05tree3+0.05tree4 | 0.84 |
| 1019-1022; 1104; 1118-1119; 1124; 1300; 1316; 1422-1423; 1460-1461 | y=2.97+0.1DFG1+0.11DFG3+0.29DFG4+0.07DFG6+0.49DFG7+0.24DFG8  +0.09DFG13+0.16DFG14+0.13DFG16-0.13tree1+0.03tree2+0.07tree3+0.05tree4 | 0.84 |
| 1024 | y=2.97+0.08DFG1+0.3DFG4+0.15DFG5+0.04DFG6  +0.79DFG7+0.13DFG9+0.09DFG15 | 0.84 |
| 1025 | y=2.97+0.1DFG1+0.26DFG4+0.15DFG5  +0.04DFG6+0.83DFG7+0.14DFG9 | 0.84 |
| 1030 | y=2.97+0.11DFG1+0.12DFG3+0.26DFG4+0.57DFG7+0.2DFG8+0.13DFG10  +0.1DFG13+0.15DFG14-0.18tree1+0.01tree2+0.12tree3+0.08tree4 | 0.84 |
| 1031; 1038; 1063; 1357; 1362 | y=2.97+0.13DFG1+0.09DFG3+0.27DFG4+0.1DFG6+0.46DFG7  +0.29DFG8+0.21DFG14-0.11tree1+0.1tree3+0.03tree4 | 0.83 |
| 1036-1037; 1196-1197; 1199; 1294; 1361; 1363; 1388; 1395-1396; 1418-1419 | y=2.97+0.09DFG1+0.11DFG3+0.26DFG4+0.1DFG6  +0.83DFG7+0.13DFG13-0.14tree1+0.1tree3 | 0.83 |
| 1039 | y=2.97+0.11DFG1+0.34DFG4+0.63DFG7+0.27DFG8  +0.11DFG15-0.07tree2+0.09tree4 | 0.83 |
| 1041; 1275; 1291; 1317-1318; 1347; 1351; 1379; 1384 | y=2.94+0.09DFG1+0.28DFG4+0.15DFG5+0.05DFG6+0.47DFG7  +0.26DFG8+0.17DFG14+0.15DFG16-0.05tree1+0.03tree2 | 0.85 |
| 1049; 1283; 1284 | y=2.97+0.09DFG1+0.28DFG4+0.15DFG5  +0.04DFG6+0.82DFG7+0.11DFG9+0.11DFG13 | 0.84 |
| 1059 | y=2.97+0.37DFG4+0.08DFG6+0.77DFG7  +0.12DFG9+0.13DFG15+0.04tree4 | 0.82 |
| 1064-1065; 1089 | y=2.97+0.08DFG1+0.29DFG4+0.16DFG5+0.05DFG6  +0.79DFG7+0.08DFG9+0.14DFG16 | 0.85 |
| 1066 | y=2.97+0.23DFG4+0.17DFG5+0.6DFG7+0.27DFG8  +0.16DFG14-0.1tree1+0.04tree3+0.04tree4 | 0.82 |
| 1069 | y=2.97+0.07DFG1+0.31DFG4+0.16DFG5+0.04DFG6  +0.78DFG7+0.06DFG9+0.1DFG13+0.13DFG16 | 0.85 |
| 1070; 1090; 1100 | y=2.97+0.07DFG1+0.31DFG4+0.16DFG5+0.04DFG6  +0.78DFG7+0.06DFG9+0.1DFG13+0.13DFG16 | 0.85 |
| 1071; 1117; 1162; 1165; 1193-1195; 1205; 1299; 1334; 1344; 1414; 1452 | y=2.93+0.1DFG1+0.11DFG3+0.29DFG4+0.07DFG6+0.49DFG7+0.24DFG8  +0.09DFG13+0.16DFG14+0.13DFG16-0.13tree1+0.03tree2+0.07tree3+0.05tree4 | 0.84 |
| 1084; 1085 | y=2.94+0.1DFG1+0.26DFG4+0.12DFG5+0.06DFG6+0.53DFG7+0.19DFG8  +0.09DFG10+0.02DFG12+0.08DFG13+0.17DFG14-0.06tree1+0.03tree2 | 0.84 |
| 1096; 1106; 1222 | y=2.94+0.33DFG4+0.11DFG6+0.48DFG7+0.22DFG8+0.19DFG14  +0.15DFG16-0.07tree1+0.02tree2+0.02tree4 | 0.83 |
| 1097; 1185; 1354-1356; 1368; 1382-1383 | y=2.93+0.1DFG1+0.09DFG3+0.29DFG4+0.09DFG6+0.46DFG7  +0.25DFG8+0.19DFG14+0.14DFG16-0.09tree1+0.05tree2+0.05tree4 | 0.84 |
| 1105 | y=2.92+0.1DFG1+0.11DFG3+0.29DFG4+0.07DFG6+0.49DFG7+0.24DFG8  +0.09DFG13+0.16DFG14+0.13DFG16-0.13tree17+0.03tree18+0.07tree19+0.05tree20 | 0.84 |
| 1110; 1219-1221 | y=2.98+0.39DFG4+0.09DFG6+0.73DFG7+  0.07DFG9+0.11DFG15+0.12DFG16+0.02tree4 | 0.83 |
| 1115; 1145-1146; 1158; 1214; 1290; 1370-1371; 1374; 1378; 1385; 1407; 1429 | y=2.97+0.08DFG1+0.29DFG4+0.15DFG5+0.05DFG6+0.5DFG7+0.24DFG8  +0.08DFG13+0.15DFG14+0.14DFG16-0.06tree1+0.03tree2 | 0.85 |
| 1116 | y=2.97+0.33DFG4+0.11DFG6+0.47DFG7+0.22DFG8+0.04DFG10  +0.19DFG14+0.13DFG16-0.09tree1-0.01tree2+0.07tree3+0.01tree4 | 0.83 |
| 1130; 1133-1136; 1144; 1148-1150; 1154-1155; 1184; 1237-1238; 1241-1247; 1250-1257; 1259; 1369 | \| y=2.99+0.51DFG16 \| \| --- \| | 0.53 |
| 1131-1132; 1147;1151; 1244; 1258; 1399 | \| y=2.98-0.34tree17 \| \| --- \| | 0.42 |
| 1137-1139; 1143; 1156; 1160; 1171; 1174; 1179; 1224; 1242-1243 | \| y=2.99+0.51DFG16 \| \| --- \| | 0.53 |
| 1140; 1142; 1159; 1170; 1172; 1178; 1201-1202; 1465 | \| y=2.99+0.51DFG16 \| \| --- \| | 0.53 |
| 1141; 1198; 1249; 1353 | \| y=2.97+0.42DFG3-0.31tree1+0.20tree4 \| \| --- \| | 0.47 |
| 1152-1153 | \| y=2.97+0.43DFG9+0.3tree16 \| \| --- \| | 0.58 |
| 1166 | y=2.97+0.1DFG1+0.11DFG3+0.29DFG4+0.07DFG6+0.49DFG7+0.24DFG8  +0.09DFG13+0.16DFG14+0.13DFG16-0.13tree1+0.03tree2+0.07tree3+0.05tree4 | 0.84 |
| 1173; 1203; 1235-1236; 1248 | \| y=2.99+0.51DFG16 \| \| --- \| | 0.53 |
| 1175; 1182-1183 | \| y=2.97+0.58DFG10+0.17DFG16 \| \| --- \| | 0.74 |
| 1187 | \| y=2.97+0.58DFG10+0.17DFG16 \| \| --- \| | 0.74 |
| 1190 | \| y=2.97+0.58DFG10+0.17DFG16 \| \| --- \| | 0.74 |
| 1227-1228 | \| y=2.97+0.31DFG4+0.09DFG60.82DFG70.14DFG90.04tree4 \| \| --- \| | 0.81 |
| 1229-1230 | y=2.97+0.28DFG4+0.7DFG7+0.28DFG8-0.14tree2+0.06tree3+0.06tree4 | 0.81 |
| 1231-1232 | y=2.92+0.1DFG1+0.09DFG3+0.29DFG4+0.09DFG6+0.46DFG7  +0.25DFG8+0.19DFG14+0.14tree1-0.09tree2+0.05tree3+0.05tree4 | 0.84 |
| 1269 | y=2.93+0.11DFG1+0.12DFG3+0.26DFG4+0.57DFG7+0.2DFG8  +0.13DFG10+0.1DFG13+0.15DFG14-0.18tree17+0.01tree18+0.12tree19+0.08tree20 | 0.84 |
| 1273 | y=2.97+0.08DFG1+0.29DFG4+0.16DFG5+0.05DFG6+0.79DFG7+0.08DFG9+0.14DFG16 | 0.85 |
| 1274 | y=2.937+0.1DFG1+0.09DFG3+0.29DFG4+0.09DFG6+0.46DFG7  +0.25DFG8+0.19DFG14+0.14DFG16-0.09tree1+0.05tree2+0.05tree4 | 0.84 |
| 1276 | y=2.94+0.11DFG1+0.23DFG4+0.17DFG5+0.67DFG7+0.29DFG8-0.08tree2+0.08tree4 | 0.83 |
| 1307; 1430 | y=2.970+0.09DFG1+0.28DFG4+0.15DFG5+0.04DFG6+0.82DFG7+0.11DFG9+0.11DFG13 | 0.84 |
| 1333 | y=2.9+0.09DFG1+0.28DFG4+0.15DFG5+0.05DFG6+0.47DFG7  +0.26DFG8+0.17DFG14+0.15DFG16-0.05tree1+0.03tree2 | 0.85 |
| 1336 | y=2.9+0.1DFG1+0.09DFG3+0.29DFG4+0.09DFG6+0.46DFG7  +0.25DFG8+0.19DFG14+0.14DFG16-0.09tree1+0.05tree2+0.05tree4 | 0.84 |
| 1337 | y=2.93+0.08DFG1+0.29DFG4+0.16DFG5+0.05DFG6  +0.79DFG7+0.08DFG9+0.14DFG16 | 0.85 |
| 1338 | y=2.95+0.09DFG1+0.27DFG4+0.16DFG5+0.65DFG7  +0.26DFG8+0.09DFG15-0.07tree2+0.08tree4 | 0.83 |
| 1364 | y=2.97+0.29DFG4+0.76DFG7+0.18DFG8+0.03DFG11  +0.14DFG13-0.14tree1+0.04tree3+0.11tree4 | 0.82 |
| 1400-1401 | y=2.97+0.37DFG3+0.22DFG11+0.04DFG12  +0.21DFG13-0.27tree1+0.02tree4 | 0.51 |
| 1415 | y=2.96+0.08DFG1+0.29DFG4+0.15DFG5+0.05DFG6+0.5DFG7+0.24DFG8  +0.08DFG13+0.15DFG14+0.14DFG16-0.06tree1+0.03tree2 | 0.85 |
| 1420 | \| y=2.30+0.14DFG13+0.49DFG16 \| \| --- \| | 0.65 |
| 1421 | \| y=2.30+0.14DFG13+0.49DFG16 \| \| --- \| | 0.65 |
| 1463 | y=2.97+0.37DFG5+0.28DFG12-0.08DFG13+0.19DFG15+0.47DFG16-0.21tree4 | 0.69 |
| 1464 | \| y=2.30+0.14DFG13+0.49DFG16 \| \| --- \| | 0.65 |
| 1469 | \| y=2.98DFG0+0.42DFG5+0.19DFG15+0.45DFG16-0.25tree4 \| \| --- \| | 0.66 |

**Note:** DFG means drought/flood grades data, tree means tree ring chronology data.
